# USP1-dependent RPS16 protein stability drives growth and metastasis of human hepatocellular carcinoma cells

**DOI:** 10.1186/s13046-021-02008-3

**Published:** 2021-06-21

**Authors:** Yuning Liao, Zhenlong Shao, Yuan Liu, Xiaohong Xia, Yuanfei Deng, Cuifu Yu, Wenshuang Sun, Weiyao Kong, Xiaoyue He, Fang Liu, Zhiqiang Guo, Guoxing Chen, Daolin Tang, Huoye Gan, Jinbao Liu, Hongbiao Huang

**Affiliations:** 1 Institute of Digestive Disease of Guangzhou Medical University, The Sixth Affiliated Hospital of Guangzhou Medical University, Qingyuan People’s Hospital, 511500 Qingyuan, Guangdong China; 2 Affiliated Cancer Hospital & institute of Guangzhou Medical University,Guangzhou Municipal and Guangdong Provincial Key Laboratory of Protein Modification and Degradation, School of Basic Medical Sciences, Guangzhou Medical University, 511436 Guangzhou, Guangdong China; 3 Department of Pathology, First People’s Hospital of Foshan, 528000 Foshan, Guangdong China; 4 Department of Surgery, UT Southwestern Medical Center, 75390 Dallas, Texas USA

**Keywords:** Hepatocellular carcinoma, USP1, RPS16, Degradation

## Abstract

**Background:**

Hepatocellular carcinoma (HCC) remains a medical challenge due to its high proliferation and metastasis. Although deubiquitinating enzymes (DUBs) play a key role in regulating protein degradation, their pathological roles in HCC have not been fully elucidated.

**Methods:**

By using biomass spectrometry, co-immunoprecipitation, western blotting and immunofluorescence assays, we identify ribosomal protein S16 (RPS16) as a key substrate of ubiquitin-specific peptidase 1 (USP1). The role of USP1-RPS16 axis in the progression of HCC was evaluated in cell cultures, in xenograft mouse models, and in clinical observations.

**Results:**

We show that USP1 interacts with RPS16. The depletion of USP1 increases the level of K48-linked ubiquitinated-RPS16, leading to proteasome-dependent RPS16 degradation. In contrast, overexpression of USP1-WT instead of USP1-C90A (DUB inactivation mutant) reduces the level of K48-linked ubiquitinated RPS16, thereby stabilizing RPS16. Consequently, USP1 depletion mimics RPS16 deficiency with respect to the inhibition of growth and metastasis, whereas transfection-enforced re-expression of RPS16 restores oncogenic-like activity in USP1-deficient HCC cells. Importantly, the high expression of USP1 and RPS16 in liver tissue is a prognostic factor for poor survival of HCC patients.

**Conclusions:**

These findings reveal a previously unrecognized role for the activation of USP1-RPS16 pathway in driving HCC, which may be further developed as a novel strategy for cancer treatment.

**Supplementary Information:**

The online version contains supplementary material available at 10.1186/s13046-021-02008-3.

## Background

Hepatocellular carcinoma (HCC) is the most common malignancy of the liver and the third leading cause of cancer deaths worldwide [1]. Patients with HCC usually do not cause any symptoms in the early stages of the disease process, thereby losing the chance of possible cure. In addition, patients with advanced HCC usually show tumor metastasis and invasion, and are prone to treatment resistance [2–4]. Indeed, although many tyrosine kinase inhibitors, such as regorafenib, sorafenib, and lenvatinib, have been developed and used clinically to treat the locally advanced or metastatic HCC, they can only extend survival by a few months [3, 5, 6]. The molecular mechanisms of the occurrence and metastasis of HCC remain to be elucidated. A deeper understanding of the underlying mechanisms will boost the discovery of biomarkers for diagnosis and become an effectively therapeutic target for the treatment of HCC.

Ubiquitin-proteasome system (UPS) is a protein degradation control system, which is ubiquitous in eukaryotes. UPS regulates various biological processes and maintains cell homeostasis by degrading specific regulatory or abnormal proteins [7, 8]. Protein ubiquitination is the initial reaction of protein degradation, and it depends on three types of enzymes, including E1, E2 and E3. Subsequently, the ubiquitin-labeled substrates are recognized and degraded by the 26 S proteasome. Protein deubiquitination, a process controlled by deubiquitinases (DUBs), is considered as the reversal reaction of ubiquitination. Generally, human DUB can accurately identify the ubiquitinated protein substrate and effectively cleave the ubiquitin chain on the substrate to enhance its stability and ensure the balance of the ubiquitination process [9]. Abnormal DUB is implicated in multiple diseases, such as cancer, cardiovascular disease, and neurologic disorder [10–12]. DUB is overexpressed in several cancers and promotes tumor development by stabilizing certain oncoproteins, such as androgen receptor, c-Myc, and Snail [13–15]. Ubiquitin specific peptidase 1 (USP1), a member of the DUBs, usually forms with a complex with USP1-associated factor 1 (UAF1, also known as WDR48) and plays a key role in promoting the development of some cancers [16–18]. For example, USP1 is highly overexpressed in HCC and predicts a poor prognosis [19]. However, the molecular mechanism of action of USP1 in HCC remains elusive.

By an unbias screening of biological mass spectrometry and further validation, we identified that ribosomal protein S16 (RPS16, the basic component of the 40 S ribosome) is a new substrate of USP1, responsible for proliferation and metastasis of HCC cells. RPS16 was previously considered to be an oncoprotein of breast cancer and gliomas by mediating resistance to doxorubicin or activating the PI3K/AKT/Snail pathway [20, 21]. In this study, we revealed that USP1 interacts with RPS16, thereby deubiquitinating and stabilizing RPS16 via its DUB activity on C90 site. Consequently, the USP1-RPS16 axis boosts the growth and metastasis of HCC cells by elevating RPS16-dependent Twist1 and Snail. The overexpression and positive correlation of USP1 and RPS16 are also observed in tumor tissues of HCC patients. Overall, this study provides critical molecular evidence for how USP1 promotes the progression of HCC, which may greatly enrich our potential targeted therapy against advanced HCC.

## Materials and methods

### Antibodies and reagents

Antibodies used in this study were shown in Table S1. ML323, a potent inhibitor of USP1 (#S7529), sorafenib (#S7397), bortezomib (#S1013), MG132 (#S2619), and cycloheximide (#S7418), an inhibitor of protein synthesis, were purchased from Selleck-chem (Houston, TX, USA).

### Cell culture

HCC cell lines (HepG2, HCCLM3, Hep3B, and Huh7) and HEK293T cell line were purchased from the American Type Culture Collection (Manassas, VA, USA). HepG2 and Hep3B cells were grown in RPMI 1640 medium (Invitrogen, Waltham, MA, USA) with 10 % fetal bovine serum (FBS). HCCLM3, Huh7, and HEK293T cells were cultured in DMEM (Invitrogen) supplemented with 10 % FBS. The cells mentioned above were all cultured in a humidified incubator at 37 °C with 5 % CO2. Short tandem repeat profiling was used to validate cell line identities.

### Transfection of plasmids and siRNAs/shRNAs

The plasmids (CMV-MCS-3FLAG-SV40-neomycin) containing full-length human USP1 (Gene ID: 7398), truncated mutants of USP1, and inactive mutant USP1-C90A, and the plasmids (CMV-MCS-HA-SV40-neomycin) containing full-length human RPS16 (Gene ID: 6217) are constructed by GeneChem (Shanghai, China). Maps of the plasmids of FLAG-USP1 and HA-RPS16 were shown (Fig.S1). The key transfection mixture, including plasmids, RPMI opti-MEM (Gibco) and lipofectamine (Invitrogen), was prepared and established as we previously reported [22]. After incubation for 15 min, the mixture was added to the cells seeded in plates or dishes, and then placed for 48 h for further analysis. For siRNA transfection, the transfection mixture, including siRNAs targeting human USP1 (Ribobio, Jiangsu, China) or RPS16 (#sc-97,200, Santa Cruz, Shanghai, China), lipofectamine RNAiMax (Invitrogen), and RPMI opti-MEM, was prepared and established as we previously reported [22]. The siRNAs sequence of human USP1 are as follows: siRNA-1: 5’-GCAGATTATGAGCTATACA − 3’; siRNA-2: 5’-GGTTGCTAGTACAGCGTTT-3’; siRNA-3: 5’-GAGAACCAGAGACAAACTA-3’. For lentivirus shRNA transfection, lentivirus (pLKD-U6-MCS-Ubiquitin-EGFP-IRES-puro-shRNA) containing 2 pairs of RNAs targeting human USP1 or non-specific sequences were purchased from GeneChem. HCC Cells were incubated with medium containing 5 µg/ml polybrene (#sc-134,220, Santa Cruz) for 15 min, and then lentiviruses were added to the cells at a multiplicity of infection of 10. After transfection for 48 h, puromycin (#S7417, Selleck-chem) was used to eliminate the unsuccessfully transfected cells at a concentration of 2 µg/ml. The sequences of human USP1 shRNAs are as follows: sh-USP1-1: 5’-ccggGCAGATTATGAGCTATACActcgagTGTATAGCTCATAATCTG Ctttttg-3’; sh-USP1-3: 5’-ccggGAGAACCAGAGACAAACTActcgagTAGTTTGTCT CTGGTTCTCtttttg-3’.

### Immunofluorescence assay

According to previous description [23], HCC cells transfected with FLAG-USP1 or HA-RPS16 plasmids, and HCC cells treated with ML323 for 48 h, were fixed with 4 % paraformaldehyde, and subjected to permeabilization with 0.5 % Triton-X for 5 min. 5 % BSA was then used to block the cells for 30 min at room temperature. Primary antibodies were added in the cells at 4 °C overnight. After wash with cold PBS for three times, the secondary antibodies were then used to react to the primary antibodies in the dark for 1 h. Finally, a mounting medium with DAPI solution (Abcam, #ab104139) was used to visualize nuclei and preserve fluorescence under a confocal microscope (Leica TCS SP8).

### Western blotting

Western blotting was used to determine protein levels. Totally proteins harvested from the HCC cells. After protein determination, 30 µg proteins were loaded on SDS-PAGE gels and then transferred onto PVDF membranes. The PVDF membranes were blocked with 5 % nonfat milk for 1 h, and then incubated with the primary antibodies at 4 °C overnight. Subsequently, the membranes were incubated with secondary antibodies at room temperature for 1 h, the ECL detection reagents (Thermo Scientific, #35,050) was used to link to the secondary antibodies and react to X-ray films. During each incubation, the membranes were washed with PBS-T for three times (5 min each time).

### Co-immunoprecipitation assay

Co-immunoprecipitation (Co-IP) was performed to explore the protein interactions on the targeted protein using an Antibody Coupling Kit (#14311D, Invitrogen) as previously reported [24]. The dynabeads were incubated with the specified antibodies for 16–24 h accordingly, and then incubated with the cell lysates extracted from HCC cells for 1–2 h. The protein-dynabeads-specified antibodies complexes were mixed with blue SDS-binding buffer, and subjected to the incubation in 70 °C water for 10 min. Subsequently, interacting proteins were separated from the complexes under the centrifugation at 13,000 rpm for 2 min. After that, the supernatant was used for further analysis, including biological mass spectrometry and western blotting.

### Cell proliferation assays

In this study, cell proliferation assays consist of cell viability determination, colony formation and EdU staining (#C10310-1, Ribobio), and were performed as previously described [25]. Cell viability assay was performed by using the MTS Kit (#G3581, Promega, Madison, WI, USA). Colony formation assay was designed to evaluate the proliferative ability of HCC cells in the long-term. Briefly, HCC cells were plated in 60 mm dishes and treated with the indicated chemicals. Post the treatment for 2 days, the cells were re-plated on a 6-well plate growing at least 2 weeks, which were visible after being stained with crystal violet. The DNA reproduction rate was evaluated by EdU assay according to standard technique. All experiments were performed at least in triplicate.

### Cell migration assay

Transwell migration assay refers to the literature that has been reported [26]. In short, HCC cells were seeded on 6-well plates overnight and were treated with ML323 for 48 h, or cells stably expressing shUSP1-1, shUSP1-3, or control shRNAs were seeded on 6-well plates for 24 h. Next, the treated cells were resuspended in serum-free medium and added to the upper chamber (#09420061, Costar, New York, USA) to make the final concentration of 1 × 10^5^ cells/well, and medium containing 10 % FBS was added into the lower chambers, then incubated for 48 h. The cells that had migrated through the middle membrane to the lower surface were fixed with 4 % paraformaldehyde for 15 min, following by staining with 1 % crystal violet solution for 5 min. All experiments were performed at least in triplicate.

### Animals

BALB/c nude mice were purchased from Charles River Laboratories (Beijing, China) and housed at the animal center of Guangzhou Medical University in adherence to ethical treatment of animals. This study strictly adheres to the ARRIVE Guidelines for reporting animal research. In short, 6-week-old male mice were housed in individually ventilated cages and fed with enough food and water in a standard room without specific pathogens. Mice were randomly divided into three groups (n = 10 per group). 2 × 10^7^ HepG2 cells stably expressing USP1 shRNA-1, shRNA-3 or control shRNAs were subcutaneously injected into each mouse. After inoculation for 27 days, BALB/c nude mice were sacrificed by cervical dislocation after CO_2_ inhalation. Tumor size, tumor weight and body weight of the mice were measured according to the previous studies [27, 28].

### Immunohistochemistry assay

HepG2 xenografts fixing, embedding and section were performed according to standard techniques. Immunohistochemistry assay was performed using a MaxVision Kit (Maixin Biol) according to the manufacturer’s instruction. The primary antibodies include anti-Ki67 and anti-RPS16 in this assay. Image quantification was performed by utilizing the Image J software. All assays were performed at least in triplicate.

### Real-Time PCR (RT-PCR) assay

General RNAs extracted from HCC cells were subjected to the RT-PCR assay as previously reported [22]. PCR primers are selected from previous reports [20, 29, 30] and listed in Table S2. All experiments were performed at least in triplicate.

### Molecular dynamics

These assays were performed as we previously described [31]. Docking analysis was performed by PatchDock (https://bioinfo3d.cs.tau.ac.il/PatchDock/). The crystal structure of USP1 (B Chains in USP1-UAF1 complex, PDB ID: 7AY0) and RPS16 (Y Chains in human 48 S translational initiation complex, PDB ID: 6YBS) were obtained from protein data bank (http://www.rcsb.org/).

### Statistical analysis

The statistical significance of data was evaluated by student’s t-tests or one-way ANOVA. To determine survival curves, the Kaplan-Meier way and ANOVA was used where appropriate. Pearson correlation analysis was used to determine the correlations between USP1 and RPS16 protein levels. SPSS 16.0 and GraphPad Prism 7.0 were applied for the analyses. A two-sided *P* value of < 0.05 was regarded as statistically significant. *P < 0.05, ^#^*P* < 0.01, ^##^*P* < 0.001, ^###^*P* < 0.0001. The statistical data are presented as mean ± SD from three independent experiments where applicable.

## Results

### RPS16 is a substrate of USP1

**Fig. 1:**
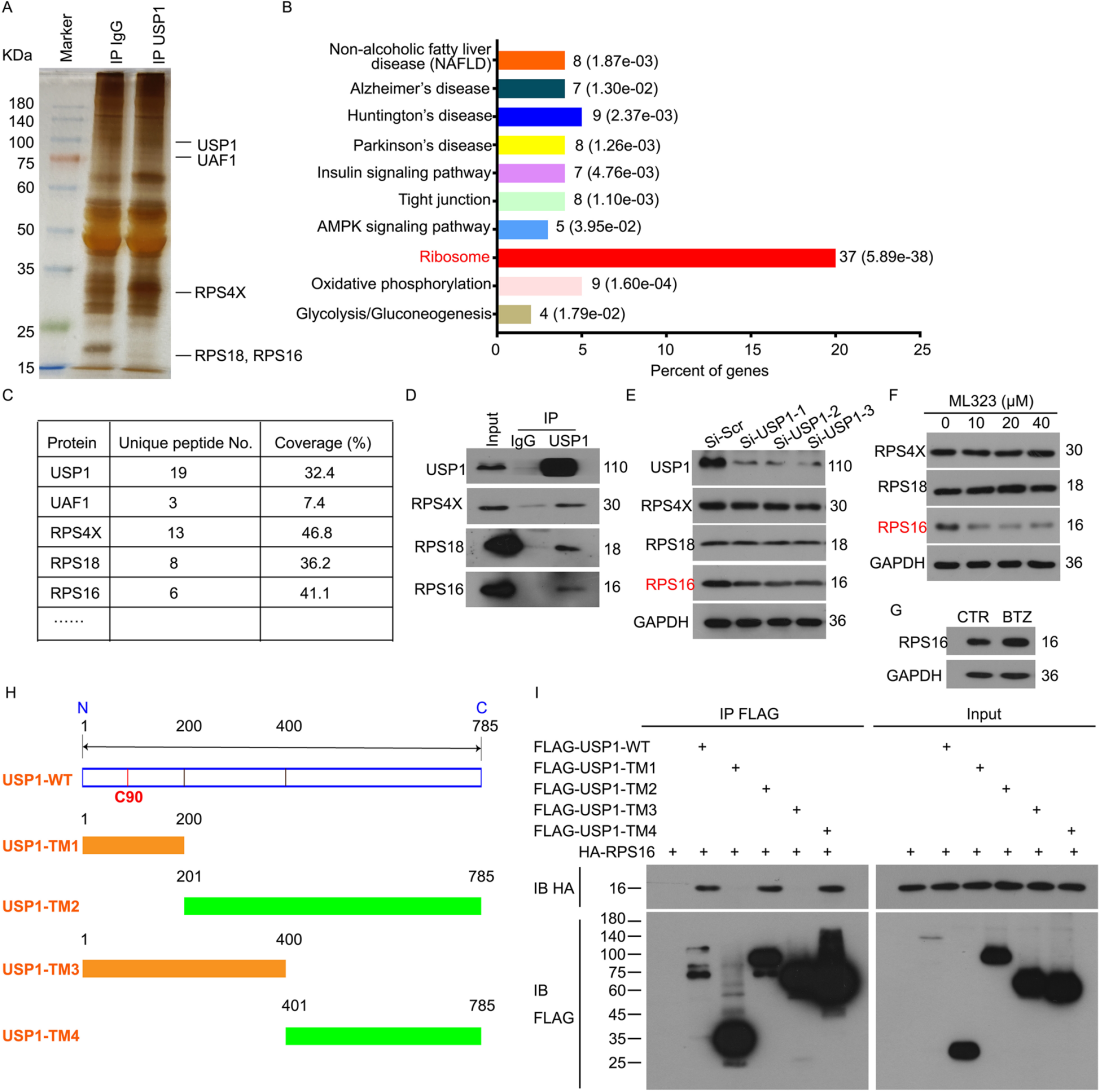
USP1 interacts with and regulates RPS16.** a** Endogenous USP1 was immunoprecipitated from HepG2 cells. The USP1-interacting proteins were separated by SDS-PAGE and were presented by silver staining assay. **b** Biological process analysis of the USP1-interacting proteins was performed. The numbers of regulated genes in pathway categories are shown. **c** Representative USP1-interacting proteins are shown in the table. **d** USP1 was immunoprecipitated from HepG2 cells and immunoblotted to RPS4X, RPS18, and RPS16. **e** HepG2 cells were treated with USP1 siRNAs or control siRNAs for 48 h. The expression levels of USP1, RPS4X, RPS18, and RPS16 were determined by western blot. GAPDH was used as a loading control. **f** HepG2 cells were treated with indicating doses of ML323 for 24 h. The expression levels of RPS4X, RPS18, and RPS16 were determined by western blot. **g** HepG2 cells were treated with bortezomib (BTZ, 50nM) for 24 h. Western blot was performed to determine the expression level of RPS16. **h** Linear structure models of wide type USP1 (USP1-WT) and its truncated mutants (USP1-TMs). **i** HEK293T cells were transfected with HA-RPS16 and FLAG-USP1-WT or FLAG-USP1-TMs. FLAG was immunoprecipitated from HEK293T cells and immunoblotted to HA and FLAG

Although USP1 contributes to liver tumorigenesis, the core substrate that USP1 drives the progression of HCC is still unknown. We first explored the potential substrates of USP1 by using co-IP assay combined biological mass spectrometry analysis in HepG2 cells with USP1 or control IgG antibodies. As a result, we unexpectedly found that USP1 may bind to 37 ribosomal proteins (RPs), especially RPS4X, RPS18, and RPS16 (Fig. 1a-c). Our immunoprecipitation and immunoblot assays further validated the protein-protein interaction of USP1-RPS4X, USP1-RPS18, or USP1-RPS16 (Fig. 1d). Next, we explored whether USP1 can regulate the protein levels of RPS4X, RPS18, and RPS16. The immunoblot analysis showed that both pharmacological (using ML323) and genetic (using RNAi) inhibition of USP1 can reduce the expression of RPS16, but not RPS4X and RPS18 (Fig. 1e and f). Of note, ML323 is a potent inhibitor by blocking the combination of USP1 and UAF1. Next, we wanted to know whether the proteasome can degrade RPS16. Bortezomib (BTZ), a specific inhibitor of proteasome, increased the protein expression of RPS16 in HepG2 cells, suggesting that RPS16 can be degraded through the ubiquitin-proteasome pathway (Fig. 1g). To further determine the binding domain between USP1 and RPS16, four truncated mutants (TM1, TM2, TM3, and TM4) of USP1 fused with FLAG-tag on their C-terminals were engineered and transfected with HA-RPS16 into HEK293T cells for 48 h (Fig. 1h). The co-IP assays showed that USP1-WT, TM2 and TM4, but not TM1 or TM3, were able to interact with RPS16 (Fig. 1i), indicating that the C-terminal (401–785 aa) of USP1 is responsible for the binding of RPS16.

**Fig. 2:**
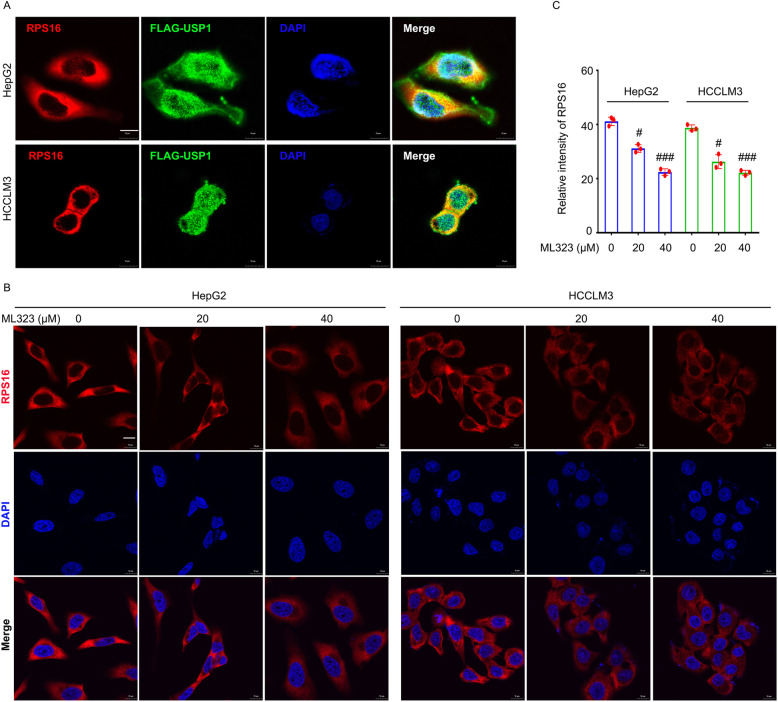
Morphological evidence of the interaction between USP1 and RPS16.** a **HepG2 and HCCLM3 cells were transfected with FLAG-USP1 plasmids for 48 h. Cell immunofluorescence assay was performed using FLAG-tag and RPS16 antibodies. **b** HepG2 and HCCLM3 cells were exposed to ML323 for 24 h. Cell immunofluorescence assay was performed using RPS16 antibodies. Representative images are shown. Scale bars, 10 μm. **c** Quantification of immunofluorescence assay in HCC cells

Additionally, by using cellular immunofluorescence assays, this study not only provided morphological evidence of USP1-RPS16 protein interaction (as shown in the yellow/orange area of the merged images) (Fig. 2a), but also showed the downregulated RPS16 level caused by the pharmacological or genetic ablation of USP1 in HCC cells (Fig. 2b, c and Fig. S2). Moreover, molecular dynamics simulation was performed to determine the interaction between USP1 and RPS16. As shown in Fig. S3a, the surface model of USP1-RPS16 complex at 50 ns was observed and established. Three-dimensional binding conformation showed that the USP1-RPS16 complex can also form (Fig. S3b). These findings provide further evidence to strengthen our hypothesis on the USP1-RPS16 complex, and may open avenues to predict compounds that inhibit the interaction between USP1 and RPS16. Collectively, we demonstrate that RPS16 is a substrate of USP1 and can be tightly regulated by the ubiquitin-proteasome pathway.

### USP1 mediates the deubiquitination and stabilization of RPS16

**Fig. 3:**
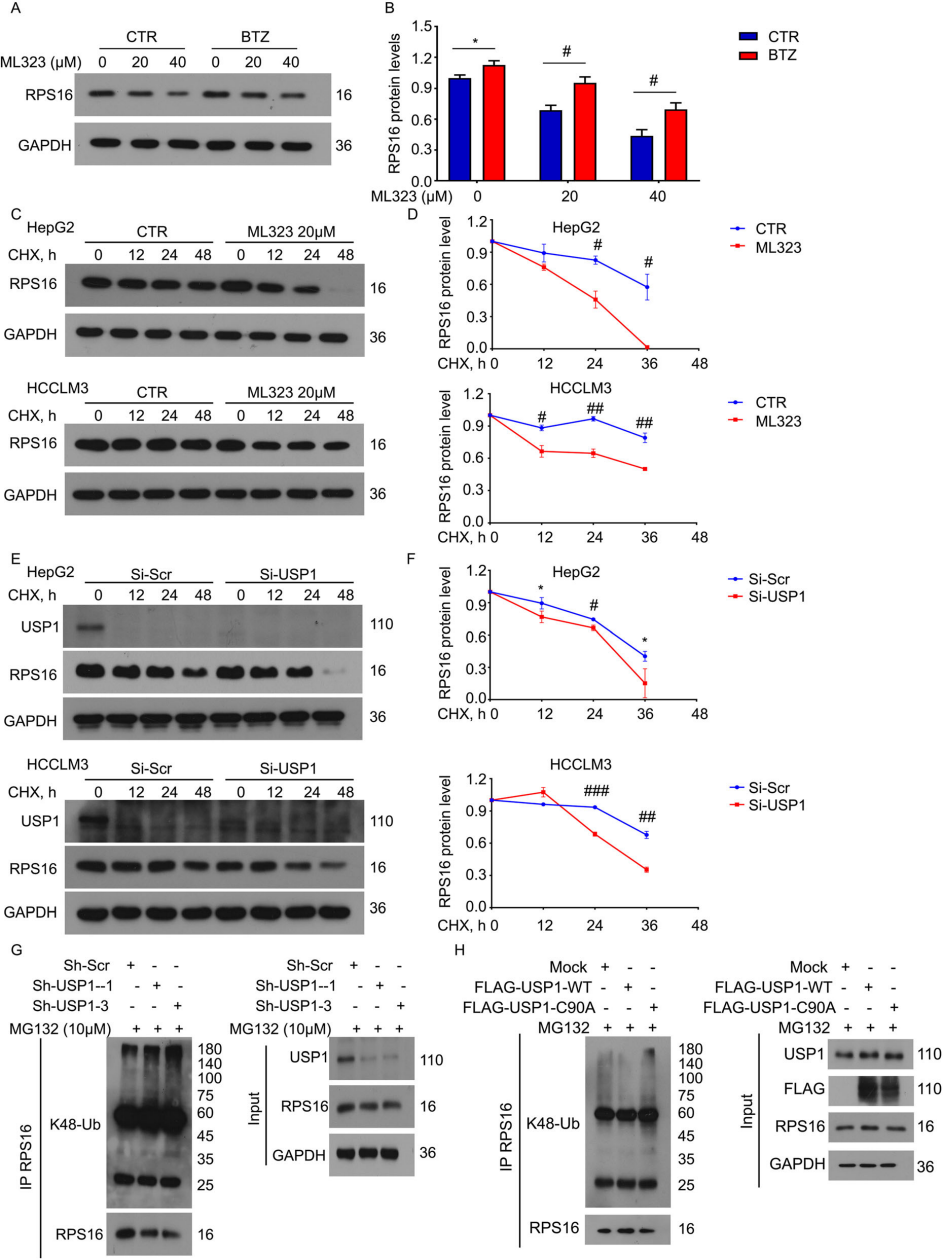
USP1 deubiquitinates and stabilizes RPS16.** a** HepG2 cells were exposed to ML323 in the absence or presence of BTZ (50 nM) for 24 h. The expression level of RPS16 was determined by western blot. **b** Quantification of the bands are shown. **c** HepG2 and HCCLM3 cells were exposed to cycloheximide (CHX, 50 µg/ml) for indicated time with or without the pretreatment of ML323 (for 24 h). The expression level of RPS16 was determined by western blot. **d** Quantification of the bands are shown. **e** HepG2 and HCCLM3 cells were exposed to CHX for indicated time with or without the pretreatment of USP1 siRNAs (for 48 h). The expression level of RPS16 was determined by western blot. **(f)** Quantification of the bands are shown. **g** RPS16 was immunoprecipitated from HepG2 cells stably expressing USP1 shRNAs or control shRNAs and immunoblotted to K48-linked ubiquitin and RPS16. MG132 (10 µM) was used to treat HepG2 cells for 6 h before harvest. **h** RPS16 was immunoprecipitated from HepG2 cells transfected with FLAG-USP1-WT, FLAG-USP1-C90A, or control plasmids, and immunoblotted to K48-linked ubiquitin and RPS16

We next wondered whether proteasome inhibition can reverse the downregulation of RPS16 induced by USP1 ablation. As we expected, the immunoblot results showed that the proteasome inhibitor BTZ rescued ML323-induced reduction of RPS16 in HepG2 cells (Fig. 3a and b). To further investigate whether USP1 can alter the protein stability of RPS16, cycloheximide (CHX) chasing analysis was performed in HCC cells following treatment with ML323 or USP1 siRNAs. Both the inhibitor and siRNAs targeting USP1 significantly shortened the half-life of RPS16, suggesting that USP1 may increase the protein stability of RPS16 (Fig. 3c-f). In contrast, the mRNA level of RPS16 was not affected by either the inhibitor or siRNAs targeting USP1 (Fig. S4), indicating that USP1 does not regulate RPS16 expression at the transcriptional level.

To confirm that USP1 is a DUB of RPS16, we further determined whether USP1 can affect the ubiquitinated level of RPS16 in HCC cells by using co-IP and immunoblot assays. The knockdown of USP1 with two independent shRNA pairs remarkably upregulated the endogenous K48-linked ubiquitination of RPS16 in HepG2 cells (Fig. 3g). To further confirm whether the DUB activity of USP1 is required for the alteration of ubiquitinated level of RPS16, the wide-type USP1 (USP1-WT) and a DUB inactive mutant of USP1 (USP1-C90A) were engineered [17] and then transfected into HepG2 cells. Subsequent Co-IP and western blotting analysis found that USP1-WT, but not USP1-C90A, downregulated the endogenous K48-linked ubiquitination of RPS16 in HCC cells (Fig. 3h). These findings demonstrate that USP1 deubiquitinates and stabilizes the ribosomal protein RPS16 through its DUB activity.

### USP1 promotes proliferation and metastasis of HCC cells

**Fig. 4:**
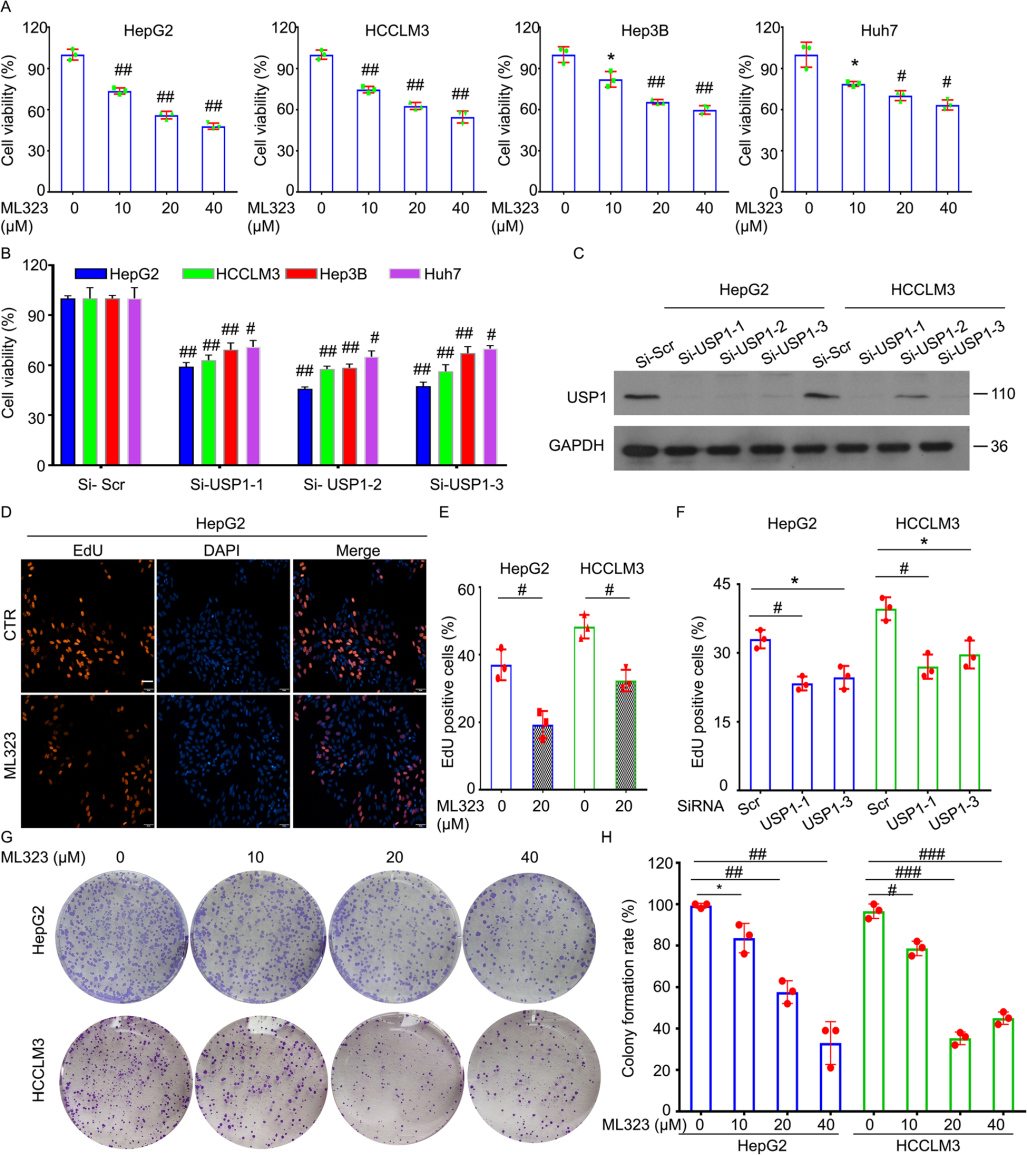
Inhibition of USP1 reduces proliferation of HCC cells.** a** HepG2, HCCLM3, Hep3B, and Huh7 cells were exposed to ML323 for 48 h. Cell viability was determined by MTS assay from three independent repeats. **b** The indicated HCC cells were treated with USP1 siRNAs or control siRNAs for 72 h. Cell viability was determined by MTS assay from three independent repeats. **c** The knockdown efficiency of USP1 was determined by western blot. **d** HepG2 and HCCLM3 cells were treated with ML323 or USP1 siRNAs for 48 h. DNA duplicate was determined by EdU staining assay. Representative images are shown. Scale bars, 50 μm. **e **and **f** Relative intensities of the images were calculated and quantified from three independent repeats. **g** HepG2 and HCCLM3 cells were treated with ML323 for 48 h. Colony formation assay was performed for 2 weeks. **h** Colony formation rate was calculated and quantified from three independent repeats

To further explore the function of USP1 in the carcinogenic phenotypes of HCC, cell viability assay was first applied to determine the proliferation of HCC cells treated with ML323 or USP1-siRNAs. Indeed, the pharmacological inhibition or the knockdown of USP1 by siRNAs reduced cell viability of various HCC cells, including HepG2, HCCLM3, Hep3B, and Huh7 (Fig. 4a-c). The EdU staining assay further confirmed that inhibiting USP1 limited DNA replication in HepG2 and HCCLM3 cells (Fig. 4d-f). The colony formation assay also showed that the long-term proliferative ability of HepG2 and HCCLM3 cells was also suppressed by the treatment of ML323 (Fig. 4g and h). Moreover, the cell viability and colony formation assays showed that the inhibition of USP1 enhanced sensitivity of HepG2 cells to sorafenib, a molecular targeted drug that treats patient with HCC (Fig. S5).

**Fig. 5:**
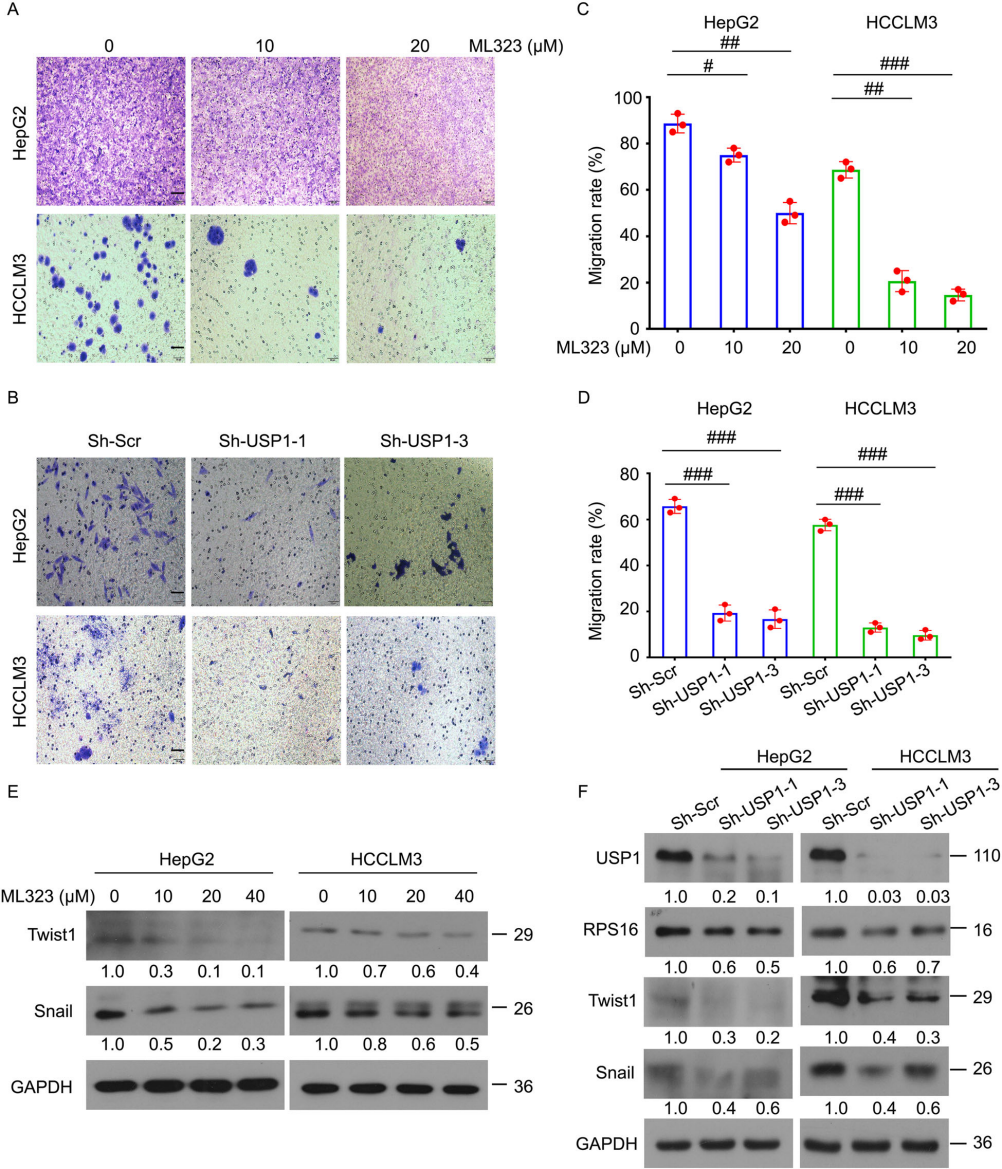
Inhibition of USP1 suppresses migration of HCC cells.** a** and **b** Transwell migration assay was performed in HepG2 and HCCLM3 cells treated with ML323, and cells stably expressing USP1 shRNAs or control shRNAs for 72 h. Representative images are shown. Scale bars, 50 μm. **c** and **d** Cell migration rate was calculated and quantified from three independent repeats. **e **and **f** Western blotting analysis was performed in HepG2 and HCCLM3 cells exposed to ML323 for 48 h, and HCC cells stably expressing USP1 shRNAs or control shRNAs using indicated antibodies. Relative intensities of the bands are shown

Most cancer (including HCC) deaths are due to metastasis [32, 33]. To investigate whether USP1 is involved in the regulation of HCC metastasis, transwell migration assay was performed in HepG2 and HCCLM3 cells exposed to ML323 or stably expressing USP1 shRNAs or control shRNAs. Inhibition of USP1 significantly reduced the migration rate of HCC cells (Fig. 5a-d). We further investigated whether USP1 regulates the expression of several key molecules (Twist1 and Snail) that promote the metastasis of HCC cells. Indeed, our immunoblotting experiments showed that the inhibition of USP1 significantly reduced the protein expression of Twist1 and Snail (Fig. 5e and f). Overall, these findings demonstrate that USP1 may motivate carcinogenic progression via promoting the proliferation and metastasis of HCC cells.

### USP1-driven cell proliferation and metastasis depends on RPS16 status

**Fig. 6:**
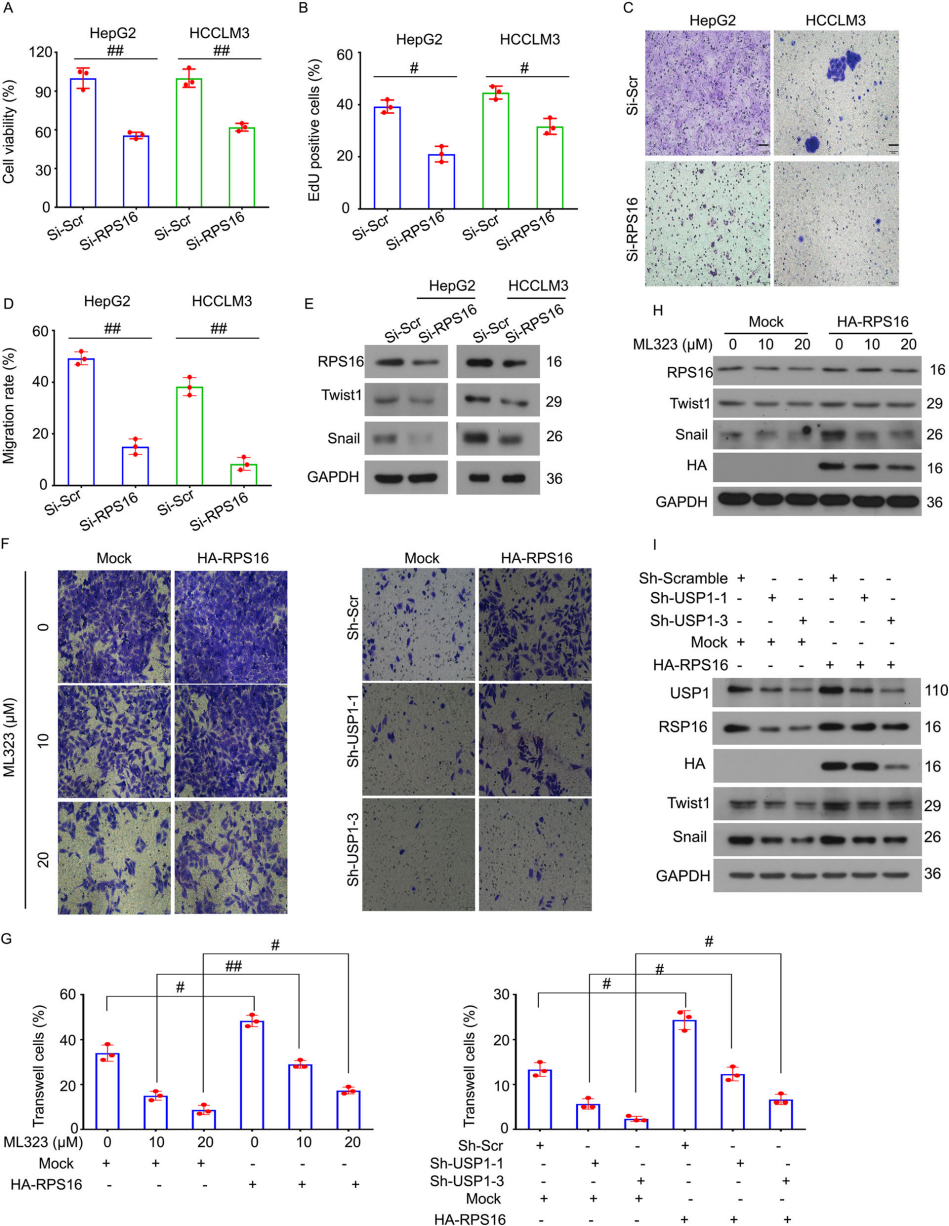
USP1-promoted cell migration depends on RPS16 status. HepG2 and HCCLM3 cells were treated with RPS16 siRNAs or control siRNAs for 72 h. **a** Cell viability was determined by MTS assay. **b** DNA duplicate was determined by EdU staining assay. **c** and **d** Cell migration rate was determined by transwell migration assay. Representative images and quantitative data are shown. **e** Expressions of RPS16, Twist1 and Snail were determined by western blotting. **f-i** HepG2 and HCCLM3 cells were treated with ML323 in the absence or presence of HA-RPS16 for 72 h, or HepG2 and HCCLM3 cells stably expressing USP1 shRNAs or control shRNAs with or without the transfection of HA-RPS16 for 72 h. **f** Cell migration was determined by transwell assays. Representative images were shown. Scale bar, 50 μm. **g** Quantification of the percentage transwell/migrated cells was shown. **h** and **i** The protein levels of RPS16, Twist1, Snail, and HA were determined by western blotting analysis

Next, we investigated whether USP1-mediated RPS16 stabilization is required for cell proliferation/metastasis in HCC cells. Cell viability, EdU staining, and transwell migration assays showed that the genetic depletion of RPS16 by siRNAs significantly inhibited proliferation and metastasis of HepG2 and HCCLM3 cells (Fig. 6a-d). Accordingly, western blot analysis showed that depletion of RPS16 significantly reduced the expression of migration-related proteins (including Twist1 and Snail) (Fig. 6e). These findings demonstrate the potential role of RPS16 in maintaining the proliferation and metastasis of HCC.

To determine whether RPS16 is a direct downstream effector responsible for USP1 function, we used HA-RPS16 in HCC cells in the absence or presence of USP1 inhibitors or shRNAs. The forced expression of RPS16 in HCC cells reversed the decrease in colony number, cell migration, as well as protein expression of Twist1 and Snail induced by inhibiting USP1 (Fig. 6f-i and Fig.S6). Together, these findings indicate that USP1-mediated RPS16 protein stability contributes to the proliferation and migration of HCC cells.

### USP1 accelerates HCC growth in vivo

**Fig. 7:**
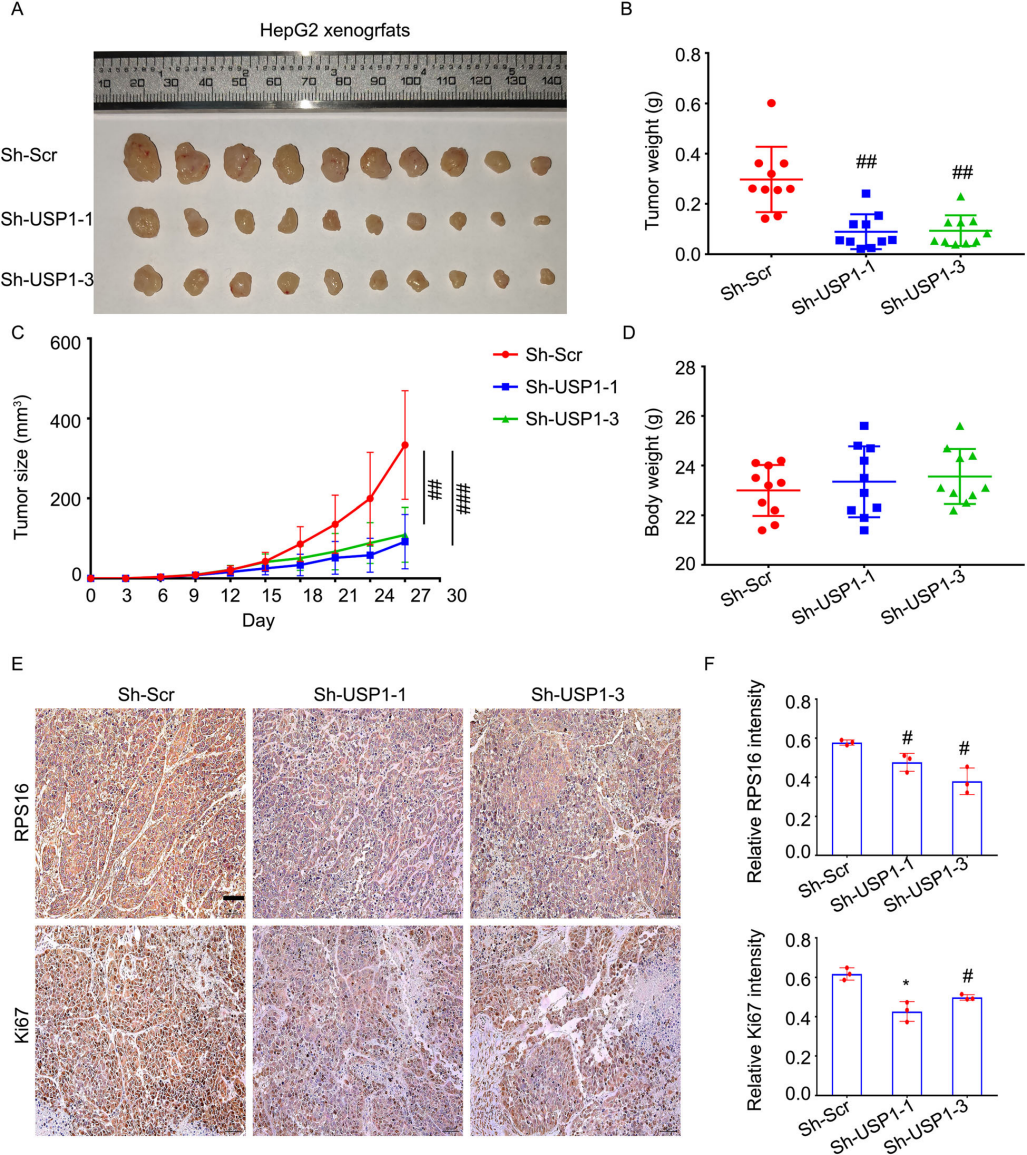
Knockdown of USP1 reduces growth of HCC *in vivo*. HepG2 cells stably expressing USP1 shRNAs or control shRNAs were transplanted in nude mice for 27 days. **a** Images of HepG2 xenografts in each group are shown. **b** Tumor weight, **c** Tumor size, and **d** body weight of nude mice is shown. **e** Expressions of RPS16 and Ki67 in tumor tissues were determined by immunohistochemistry assay. Scale bar, 50 μm. **f** Relative intensities of RPS16 and Ki67 were calculated and quantified using an Image J software

Based on in vitro studies, we further explored the function of USP1 *in vivo* by establishing a nude mice model bearing HepG2 xenografts stably expressing USP1 shRNAs or control shRNAs. In fact, compared with xenografts stably expressing control shRNA, xenografts stably expressing USP1 shRNA had significantly reduced tumor size and weight (Fig. 7a-c). Meanwhile, there was no difference of body weight among the three groups (Fig. 7d). Moreover, our immunohistochemistry assays showed that the expression of RPS16 and Ki67 in xenografts stably expressing USP1 shRNAs was lower than that in the control shRNA group (Fig. 7e and f). These animal studies established the role of USP1 in promoting tumor growth and proliferation in HCC cells.

### Clinical relevance of USP1 and RPS16 in HCC

**Fig. 8:**
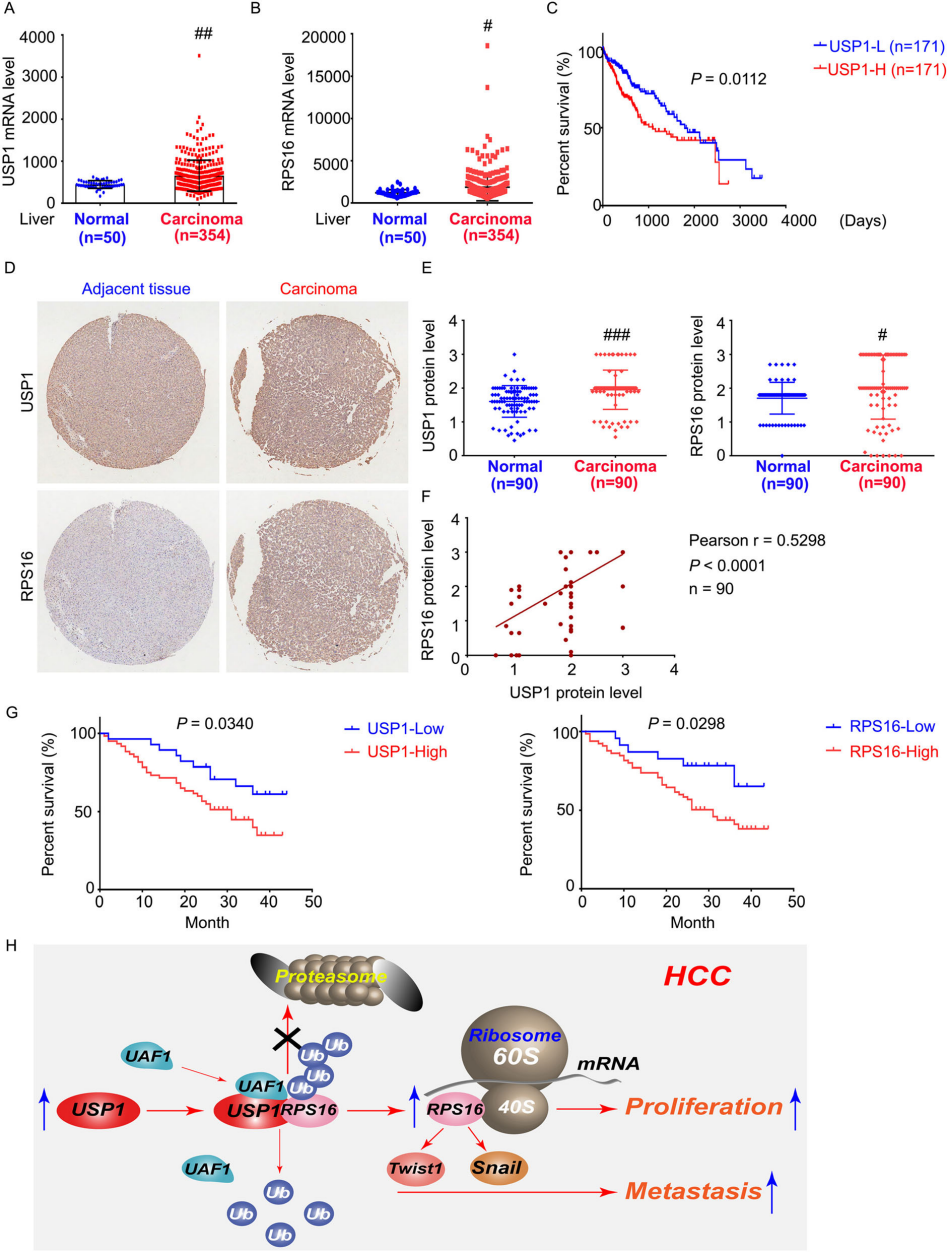
Clinical relationship between USP1 and RPS16.** a** and **b** Expressions of USP1 and RPS16 at mRNA level in HCC in comparison with normal tissues from the TCGA database. **c** Kaplan-Meier curves from patients with HCC expressing low and high USP1 from the TCGA database. **d** Immunohistochemistry assay was performed in HCC and normal adjacent tissue microarray (*n* = 90). Representative images of USP1 and RPS16 are shown. **e** Quantification of the USP1 and RPS16 expression in tissues. **f** The correlation of USP1 and RPS16 protein levels in human HCC. **g** Kaplan-Meier curves from patients with HCC expressing low and high USP1/RPS16 from the tissue microarray. **h** Graphical summary of USP1-regulated proliferation and metastasis of HCC

Finally, we explored the significance of the expression of USP1 and RPS16 in patients with HCC. We first utilized the public TCGA database (https://tcga-data.nci.nih.gov/) to analyze the mRNA levels of USP1 and RPS16 in HCC tissues. Compared with 50 normal liver tissues, USP1 and RPS16 were up-regulated in 354 HCC tissues (Fig. 8a and b). In addition, Kaplan-Meier curve analysis showed that HCC patients with higher USP1 mRNA expression had worse overall survival and prognosis compared with patients with lower USP1 expression (Fig. 8c). These mRNA data indicate that USP1 may be a biomarker for predicting poor prognosis.

In addition, we analyzed the protein levels of USP1 and RPS16 in two tissue arrays containing cancer tissues and adjacent normal tissues from 90 HCC patients (obtained from Shanghai Outdo Biotech Company). Consistent with the analysis of the TCGA database, the protein expressions of USP1 and RPS16 in liver cancer tissues are up-regulated compared with adjacent liver tissues (Fig. 8d and e). The protein level of USP1 was positively correlated with the protein level of RPS16 (Fig. 8f). Importantly, patients with higher USP1 or RPS16 protein levels had a lower overall survival rate than those with lower USP1/RPS16 protein levels (Fig. 8g). These clinical investigations potentially support our preclinical findings that USP1 regulates the deubiquitination and stabilization of RPS16 to drive the progression of HCC.

## Discussion

HCC is the most common liver cancer with a high incidence and poor clinical prognosis [1]. Due to the lack of early diagnosis methods and hallmarks, most patients diagnosed with HCC are already in the middle or late stage and are considered incurable. Although we have made great progress in targeted therapy or systemic therapy that mainly based on sorafenib, regorafenib and other tyrosine kinase inhibitors, the efficacies of these therapies are extremely limited [6, 34–36]. Therefore, we need to further understand the pathogenesis of HCC. In this study, we documented that USP1 is an oncoprotein, which is essential for the progression of HCC by stabilizing the ribosomal protein RPS16. USP1 can recognize and bind RPS16 by its C-terminal (401–785 aa). The stability of RPS16 induced by USP1 requires UAF1 (a cofactor of USP1) and the cys90 (C90) site at its N-terminal. Our study also gained a novel view on the proliferation and metastasis of liver cancer mediated by USP1-RPS16-Twist1/Snail. In addition, we showed clinical evidence of up-regulation and positive correlation of USP1 and RPS16 in HCC specimens. The discovery of the USP1-RPS16-Twist1/Snail axis may provide a potential therapeutic target for HCC and lead to more innovative drug development based on DUB inhibition.

Ribosomes have the function of synthesizing proteins and are composed of 4 RNAs and about 80 RPs in eukaryotic cells. Recently, increasing studies have been shown that the mutation and overexpression of RPs are closely related to the development of some malignant tumors [37, 38]. In particular, RPs are involved in the regulation of p53, NF-κB and other tumor-related pathways [39, 40], suggesting that RPs may exert additional biological functions beyond protein synthesis. In this study, we showed that both mRNA and protein levels of RPS16 were overexpressed in HCC. This up-regulation of RPS16 promotes the growth and migration of HCC cells by promoting the expression of Twist1 and Snail, thereby revealing the new pathological function of RPS16 in cancer.

DUB is a group of enzymes that can remove ubiquitin chains on multiple substrates, thereby mediating various biological processes. Dysregulation of the DUB is implicated in the occurrence and progression of cancers [15]. Our current research showed that USP1 promotes the growth and migration of liver cancer cells by maintaining the stability of RPS16 protein. First, USP1 ablation leads to the downregulation of RPS16 at protein level, but not at mRNA level. Additionally, the USP1 inhibitor-induced the downregulation of RPS16 is reversed by proteasome inhibitors. CHX-tracking assays further confirm that USP1 ablation shortens the protein halflife of RPS16. Second, our co-IP assays showed that USP1 potently interacts with RPS16 by its C-terminal (401–785 aa) and the suppression of USP1 expression increases ubiquitination level of RPS16. In contrast, the overexpression of USP1-WT, but not DUB inactive form of USP1, decreases ubiquitination level of RPS16. Third, the reinforced expression of RPS16 reverses the USP1 ablation-induced growth and migration of HCC cells, and their downstream metastatic effectors, including Twist1 and Snail. Finally, the analysis of clinical samples showed that the level of USP1 protein in HCC specimens is positively correlated with the level of RPS16 protein. These findings collectively demonstrate that USP1 mediates deubiquitination and stability of RPS16. This study also unravels a potential relationship between UPS and ribosome and implicates this connection in cancer research.

High proliferation and metastasis are common features that lead to recurrence and mortality of many deadly cancers. The biological processes involved in proliferation and metastasis are dominated by key players that might also be tightly regulated by UPS and ribosome. In the current study, we found that ablation of the USP1-RPS16 axis markedly reduces proliferation of HCC cells. Additionally, the USP1-RPS16 axis is required for metastasis by increasing the expression of Twist1 and Snail, two well-studied masters of cancer metastasis via activating epithelial-mesenchymal transition (EMT) [41–43]. It has been demonstrated that Twist1 functions as a transcriptional factor to activate EMT via regulating multiple downstream genes, such as Foxoa1, E-cadherin, vimentin, and slug in various invasive cancers [42–44]. The upregulation of Snail was also observed in invasive cancer cells. But unlike Twist1, Snail mechanically functions as a transcription repressor to induce EMT by inhibiting expression of E-cadherin [45, 46]. This research potentially improves our understanding of the molecular mechanisms of liver cancer proliferation and metastasis, which may further promote the prevention and treatment of fatal metastatic cancer. However, this study has a couple of limitations. On the one hand, only one type of animal model was established to evaluate the role of USP1-RPS16 axis *in vivo*; on the other hand, further research is needed to explore how the USP1-RPS16 axis changes the expression of Twist1 and Snail. There is also an urgent need to develop an effective inhibitor for USP1-RPS16 axis, and evaluate its effect on HCC proliferation and metastasis in preclinical and clinical studies.

## Conclusions

In summary, we demonstrated that the USP1-RPS16 axis favors the growth and metastasis of HCC cells (Fig. 8h). Targeting USP1-mediated RPS16 stabilization may provide a potential novel strategy for advanced HCC.

## Supplementary Information

**Additional file 1: Figure S1.** Maps of plasmids used in this study. (a) and (b) Maps of the plasmids of FLAG-USP1 and HA-RPS16 were shown, respectively. **Figure S2.** Morphological evidence of USP1 increases expression of RPS16 in the cytoplasm. Immunofluorescence assay was performed using RPS16 antibodies in HepG2 cells stably expressing USP1 shRNAs or control shRNAs. **Figure S3.** Molecular simulations for the interaction of USP1 with RPS16. (a) Surface presentation of the USP1-RPS16 complex crystal structure. (b) Three dimensional crystal structure of USP1-RPS16 complex. **Figure S4.** USP1 does not alter RPS16 mRNA expression. (a) and (b) mRNA levels of indicated molecule were determined by qRT-PCR assay in HepG2 cells treated with ML323 for 12 h, and cells stably expressing USP1 shRNAs or control shRNAs. **Figure S5.** Inhibition of USP1 increases sensitivity of HepG2 cells to sorafenib. (a) and (b) HepG2 cells were exposed to sorafenib with or without ML323 for 48 h. Cell viability was determined by MTS assay. Long-term proliferative ability was determined by colony formation assay for 14 days. Representative images and quantification of the colonies are shown. **Figure S6.** USP1-promoted cell proliferation depends on RPS16 status. (a) and (b) Colony formation analysis of HepG2 cells treated with ML323, or cells stably expressing USP1 shRNAs with or without HA-RPS16. (c) and (d) Quantification of (a) and (b). **Table S1.** Detailed information of primary antibodies used in this study. **Table S2. **Detailed information of primers used in this study.

## Data Availability

All the data and material supporting the conclusions were included in the main paper.

## Abbreviations

HCC: Hepatocellular carcinoma
DUBs: Deubiquitinating enzymes
USP1: Ubiquitin-specific peptidase 1
RPS16: Ribosomal protein S16
UPS: Ubiquitin-proteasome system
Co-IP: Co-immunoprecipitation
RT-PCR: Real-Time PCR
RPs: Ribosomal proteins
BTZ: Bortezomib
CHX: Cycloheximide
EMT: Epithelial-mesenchymal transition
