# Supplementary material for: USP1-dependent RPS16 protein stability drives growth and metastasis of human hepatocellular carcinoma cells
Source: J Exp Clin Cancer Res. 2021 Jun 21;40:201. doi: 10.1186/s13046-021-02008-3 (PMC8215741; doi:10.1186/s13046-021-02008-3)
Supplement: Supplementary file 1 — Additional file 1: Figure S1. Maps of plasmids used in this study. (a) and (b) Maps of the plasmids of FLAG-USP1 and HA-RPS16 were shown, respectively. Figure S2. Morphological evidence of USP1 increases expression of RPS16 in the cytoplasm. Immunofluorescence assay was performed using RPS16 antibodies in HepG2 cells stably expressing USP1 shRNAs or control shRNAs. Figure S3. Molecular simulations for the interaction of USP1 with RPS16. (a) Surface presentation of the USP1-RPS16 complex crystal structure. (b) Three dimensional crystal structure of USP1-RPS16 complex. Figure S4. USP1 does not alter RPS16 mRNA expression. (a) and (b) mRNA levels of indicated molecule were determined by qRT-PCR assay in HepG2 cells treated with ML323 for 12 h, and cells stably expressing USP1 shRNAs or control shRNAs. Figure S5. Inhibition of USP1 increases sensitivity of HepG2 cells to sorafenib. (a) and (b) HepG2 cells were exposed to sorafenib with or without ML323 for 48 h. Cell viability was determined by MTS assay. Long-term proliferative ability was determined by colony formation assay for 14 days. Representative images and quantification of the colonies are shown. Figure S6. USP1-promoted cell proliferation depends on RPS16 status. (a) and (b) Colony formation analysis of HepG2 cells treated with ML323, or cells stably expressing USP1 shRNAs with or without HA-RPS16. (c) and (d) Quantification of (a) and (b). Table S1. Detailed information of primary antibodies used in this study. Table S2. Detailed information of primers used in this study. [file 13046_2021_2008_MOESM1_ESM.docx]

**
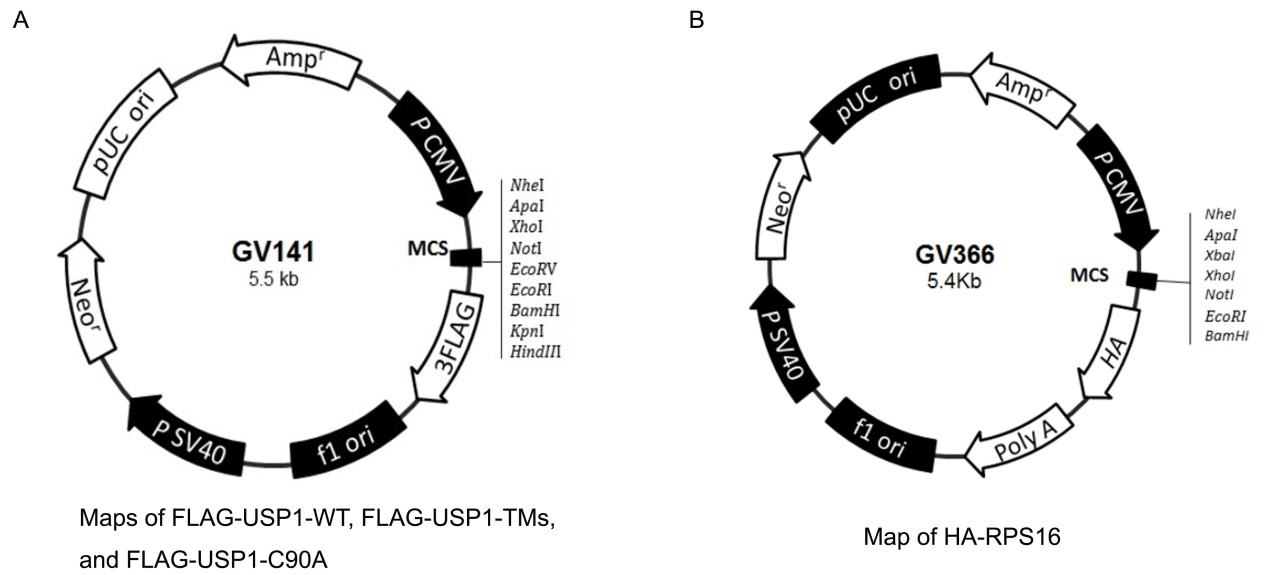
**

**Fig. S1 Maps of plasmids used in this study. (a)** and **(b)** Maps of the plasmids of FLAG-USP1 and HA-RPS16 were shown, respectively.


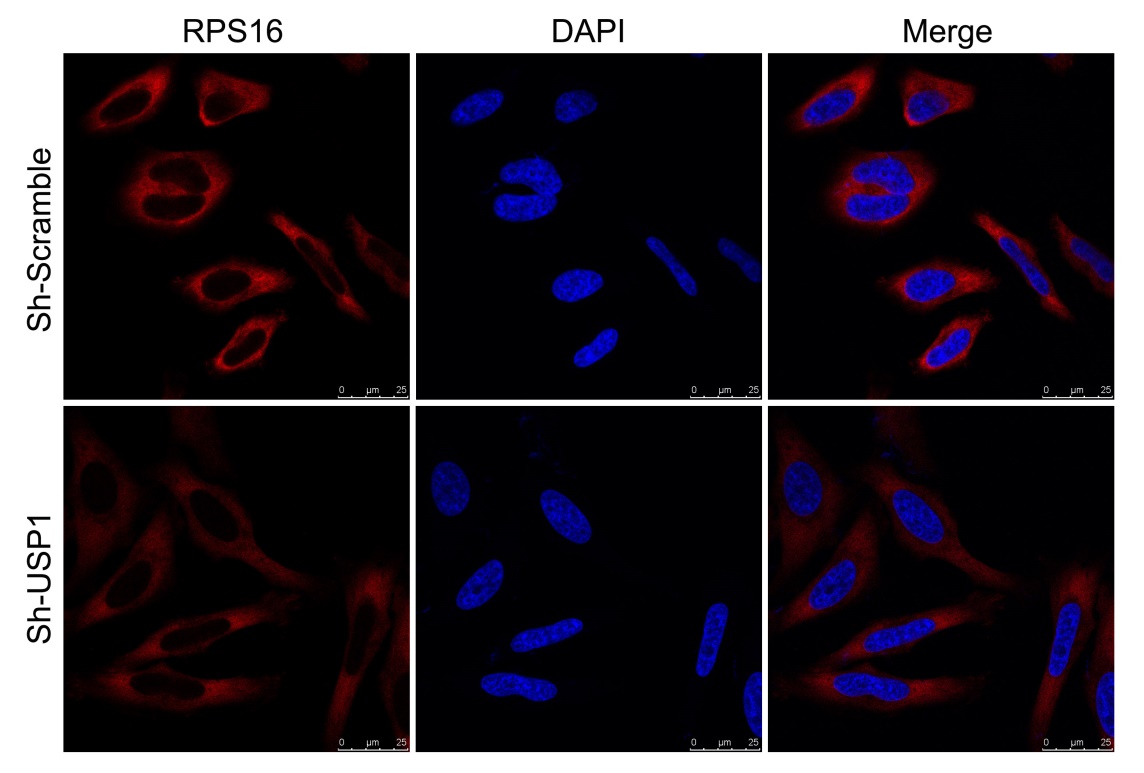


**Fig. S2 Morphological evidence of USP1 increases expression of RPS16 in the cytoplasm.** Immunofluorescence assay was performed using RPS16 antibodies in HepG2 cells stably expressing USP1 shRNAs or control shRNAs.


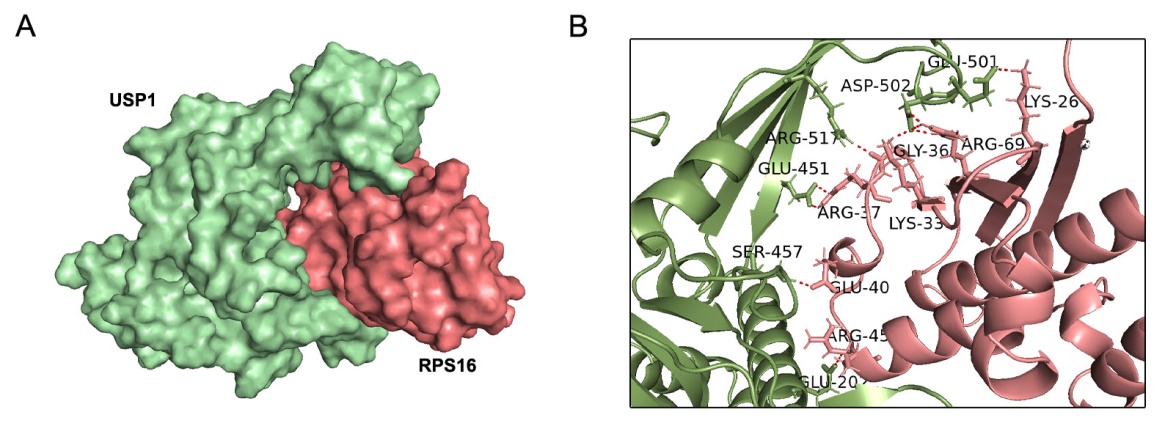


**Fig. S3 Molecular simulations for the interaction of USP1 with RPS16.** **(a)** Surface presentation of the USP1-RPS16 complex crystal structure. **(b)** Three dimensional crystal structure of USP1-RPS16 complex.

**
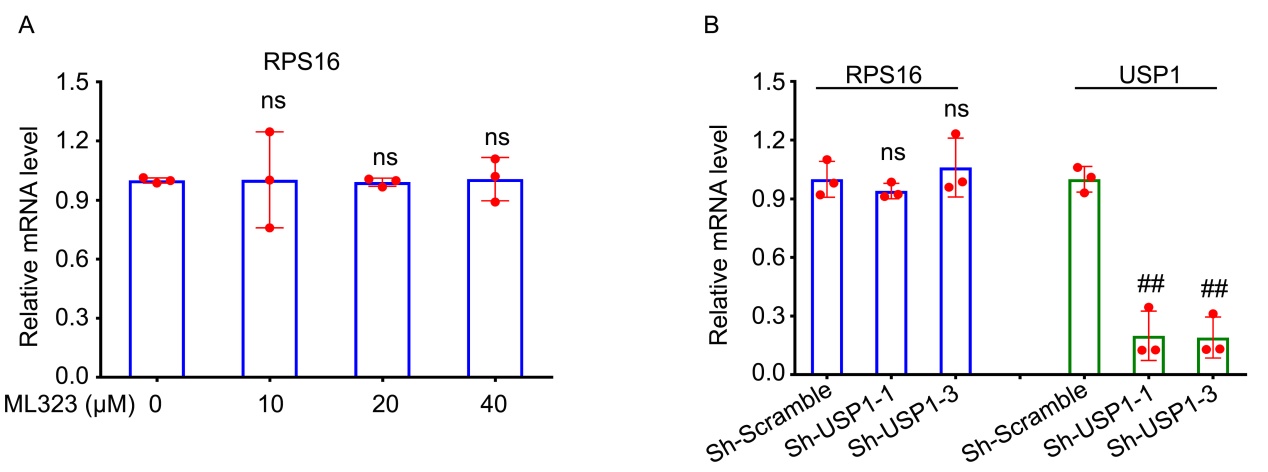
**

**Fig. S4 USP1 does not alter RPS16 mRNA expression. (a)** and **(b)** mRNA levels of indicated molecule were determined by qRT-PCR assay in HepG2 cells treated with ML323 for 12 h, and cells stably expressing USP1 shRNAs or control shRNAs.


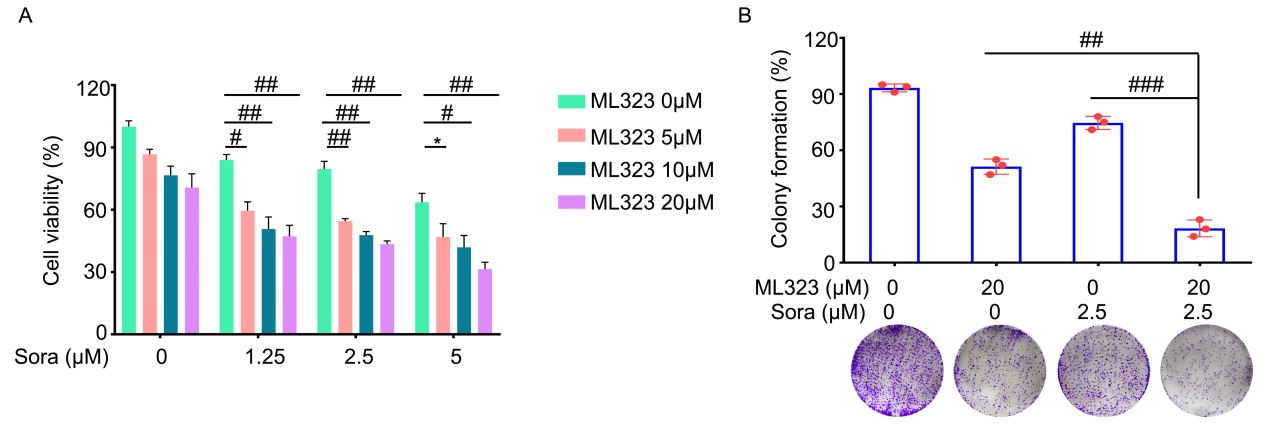


**Fig. S5 Inhibition of USP1 increases sensitivity of HepG2 cells to sorafenib. (a)** and **(b)** HepG2 cells were exposed to sorafenib with or without ML323 for 48 h. Cell viability was determined by MTS assay. Long-term proliferative ability was determined by colony formation assay for 14 days. Representative images and quantification of the colonies are shown.


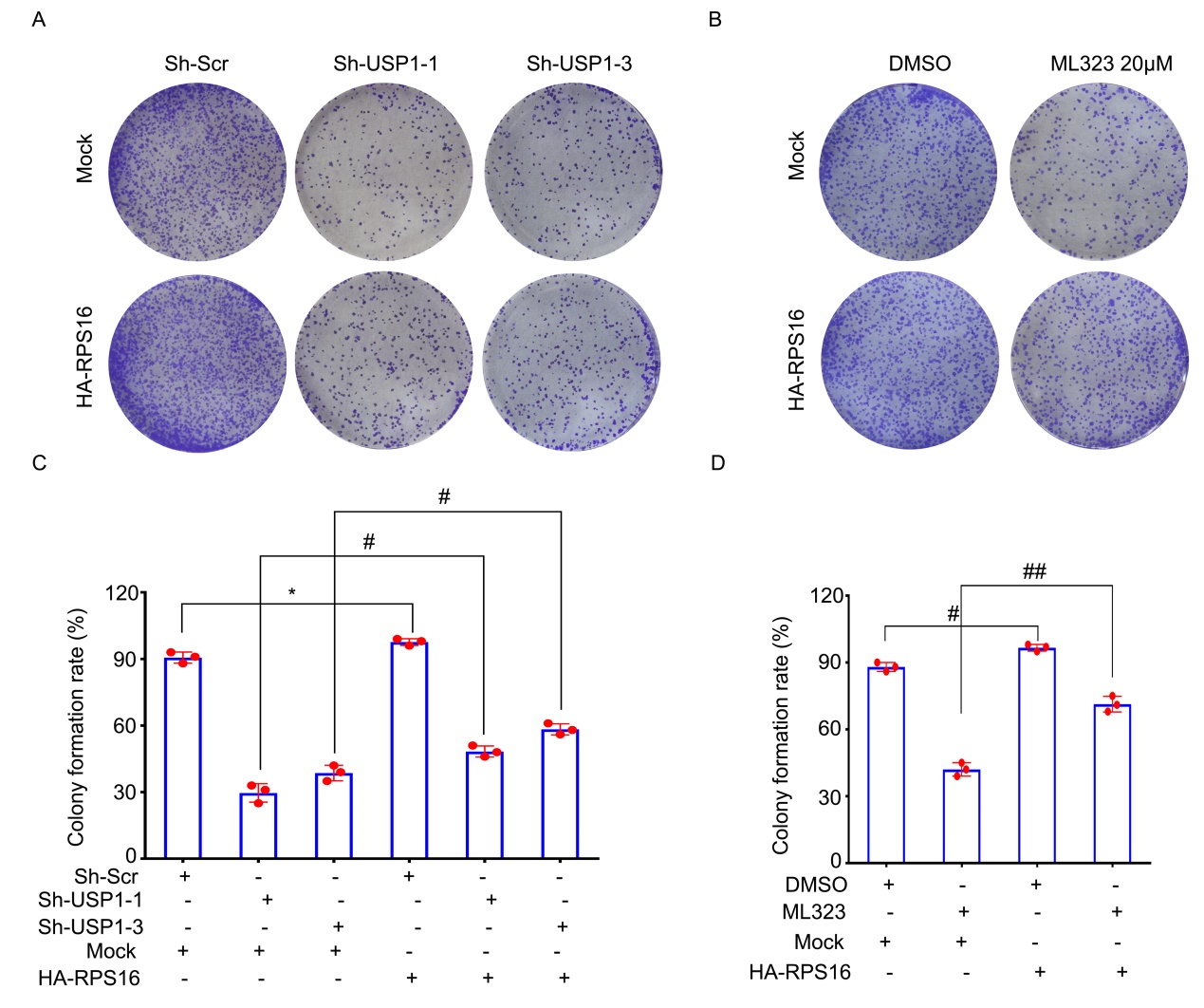


**Fig. S6 USP1-promoted cell proliferation depends on RPS16 status. (a)** and **(b)** Colony formation analysis of HepG2 cells treated with ML323, or cells stably expressing USP1 shRNAs with or without HA-RPS16. **(c)** and **(d)** Quantification of **(a)** and **(b)**.

**Table S1.** **Detailed information of primary antibodies used in this study.**

| **Primary antibodies** | **Company** | **Catalog number** | **Application** | **Dilution** |
| --- | --- | --- | --- | --- |
| anti-RPS16 | Abcam | #ab177951 | WB | 1:1000 |
| anti-RPS4X | Abcam | #ab211427 | WB | 1:1000 |
| anti-RPS18 | Abcam | #ab224519 | WB | 1:1000 |
| anti-RPS10 | Abcam | #ab151550 | WB | 1:1000 |
| anti-USP1 | Cell Signaling Technology | #8033 | WB | 1:1000 |
| anti-K48-linkage Specific Polyubiquitin | Cell Signaling Technology | #8081 | WB | 1:1000 |
| anti-Snail | Cell Signaling Technology | #3879 | WB | 1:1000 |
| anti-FLAG | Cell Signaling Technology | #8146 | WB | 1:1000 |
| anti-HA | Cell Signaling Technology | #3724 | WB | 1:1000 |
| anti-GAPDH | Cell Signaling Technology | #5174 | WB | 1:1000 |
| anti-Twist 1 | Bioworld Technology | #AP0020 | WB | 1:1000 |
| anti-RPS16 | Abcam | #ab177951 | IP | 1:50 |
| anti-USP1 | Cell Signaling Technology | #8033 | IP | 1:50 |
| anti-FLAG | Cell Signaling Technology | #8146 | IP | 1:50 |
| anti-IgG | Cell Signaling Technology | #3900 | IP | 1:50 |
| anti-RPS16 | Abcam | #ab177951 | IF | 1:200 |
| anti-FLAG | Cell Signaling Technology | #8146 | IF | 1:800 |
| anti-RPS16 | Abcam | #ab177951 | IHC | 1:100 |
| anti-USP1 | Abcam | #ab84772 | IHC | 1:100 |
| anti-Ki67 | Cell Signaling Technology | #9449 | IHC | 1:200 |

**Table S2. Detailed information of primers used in this study.**

| Gene | RPS16 | USP1 | GAPDH |
| --- | --- | --- | --- |
| Primer forward | 5′-GTGTCCGT  GTAAAGGGT  GG-3′ | 5′-CCAATGAG  AGCGGAAGG  AGG-3′ | 5′-TCCCATCAC  CATCTTCCA -3′ |
| Primer reverse | 5′-ACTGGATG  AGGATGTCTT  TGATC-3′ | 5′-CACCAATT  ATATCTAGAC  CAAAGCC-3′ | 5′-CATCACGCCA  CAGTTTCC-3′ |
| Accession number | NM_001020.6 | NM_001017415.2 | NM_002046.7 |
| Amplicon size | 138 bp | 151 bp | 380 bp |
| Amplified target | Exon spanning | Non-exon spanning | Exon spanning |
